# Zambian indigenous chicken genetic resources: phenotypic characteristics and their production systems among small-scale farmers

**DOI:** 10.3389/fvets.2024.1429869

**Published:** 2024-07-29

**Authors:** Simushi Liswaniso, Kabemba Mwambilwa, Kolawole Odubote, Thobela Louis Tyasi, Mwape Mweni, Xue Sun, Rifu Xu, Ning Qin

**Affiliations:** ^1^Department of Animal Breeding, Genetics and Reproduction, College of Animal Science and Technology, Jilin Agricultural University, Changchun, Jilin, China; ^2^Modern Agricultural Technology International Cooperative Joint Laboratory of the Ministry of Education, Changchun, China; ^3^Department of Livestock Development, Lusaka, Zambia; ^4^Department of Animal Sciences, School of Agricultural Sciences, University of Zambia, Lusaka, Zambia; ^5^Department of Agricultural Economics and Animal Production, University of Limpopo, Polokwane, South Africa

**Keywords:** body measurements, correlation analysis, health management practices, marketing practices, principal component analysis, reproductive performance

## Abstract

**Introduction:**

Indigenous chickens are very important to households for income and protein. However, their performance is usually poor, especially under small-scale farmer management, despite their potential to perform better. The performance of these chickens can be improved by selective breeding. However, for this to be a success, there is a need to understand the phenotypic and production characteristics of these chickens fully. Hence, this study aimed to characterize the phenotypes of these chickens and their production system among small-scale farmers.

**Method:**

A structured questionnaire was administered to 177 small-scale farmers. A total of 538 chickens whose mean weight was 1.66 kg were individually phenotyped in Luapula, Muchinga, and Northern provinces of Zambia.

**Results:**

Ownership of the indigenous chickens was dominated by females (65.37%), with most (64.31%) having attained primary education. Most housed their chickens in family houses (42.03%). All the farmers let their chickens scavenge for their feed, with 45.58% of them providing basic supplementation. Most (84.10%) farmers bought their breed stock from within their community and had a mean flock size of 12.5 chickens/household, which they mostly (78.09%) kept as free-range. The majority (77.39%) practiced culling, with low productivity being the most common reason for culling (84.45%). Only 59.01% of farmers practiced selective breeding, while 86.22% practiced uncontrolled mating. The age at first mating for cocks and hens was 6.8 months and 6.34 months, respectively, with 6.73 months being the age at first egg. It takes 15.43 days to reach a mean clutch size of 13 eggs. The hatchability and mortality at 8 weeks were 83.44% and 67.57%, respectively. All chickens were sold as live chickens, and the majority (51.59%) of the farmers sold their chickens within the community at 7.23 months. Diseases and predators were the most common challenges affecting farmers in the study area. Consultations with veterinarians, vaccinations, and deworming were uncommon while treating sick chickens mostly using ethnoveterinary medicines was common. The most common qualitative traits were brown (27.88%) and mixed (26.77%) plumage color, white skins (91.45%) and shanks (48.70%), single comb type (91.08%), red earlobe (55.76%), and orange eyes (78.07%). All linear body measurements positively and significantly correlated with the body weight averaging 1.66kgs, an indicator that selection for any of them would result in a corresponding increase in body weight. Principal Component Analysis extracted two components with 69.38% of the total variation.

**Discussion:**

The diversity in phenotypes of these chickens and their production systems indicate huge potential for improvement by implementing breeding programs.

## 1 Introduction

The production of indigenous chickens at the rural household level is one of the most common ventures rural communities prefer. This is because indigenous chickens help alleviate hunger and poverty at the household level (1–3). Indigenous chickens are common among small-scale rural households because they are tolerant to most diseases, withstand harsh conditions, and grow relatively quickly (1, 3, 4).

Indigenous chickens are the most produced livestock in Zambia. Zambia has 21 million indigenous chickens, and almost 1.6 million households are involved in producing them (5). However, despite these chickens being widespread, their performances are very poor as they record very poor hatchability and have higher mortalities under small-scale farmer management (1, 6). It has been reported that late maturity, low egg weights, small clutch, and small body sizes characterize the performance of indigenous chickens (7, 8).

As previously stated, indigenous chickens significantly offer local populations a less expensive source of high-quality protein (9). They are essential to maintaining food security. These indigenous chickens' peculiar taste makes them preferred among households (10). Other than being kept for food, in some parts of Africa, these chickens are farmed for different reasons, such as cultural and religious purposes (11). In some cultures, chickens are used as symbols of appreciation (10). They are a source of income for resource-constrained local populations who depend on them despite producing less meat and eggs than commercial chickens (12). Economically, these chickens contribute to household income generation, fetching as high as USD 7.22 per chicken when sold, as Gunya et al. (13) reported.

Despite the highlighted advantages and potential these chickens have to perform well in the tropics, they have struggled to meet the nutritional requirements of the growing populations due to their poor performance compared to the exotic chickens. Nonetheless, their performance can be improved through breeding programs to enhance the traits of economic importance.

For these breeding programs to be effective, these chickens must first be characterized (14). The first step in characterizing chickens involves identifying poultry populations based on their physical traits that can be useful for breeding and selection (15). However, breeding programs are specific to production systems that vary from one location to another. Therefore, characterizing the chickens as a way of understanding the variability in a particular area and understanding the production systems is also crucial.

There is limited information on Zambian indigenous chickens' phenotypic and production system characteristics. Therefore, this study aimed to phenotypically characterize Zambian indigenous chickens and their production systems among small-scale farmers in Luapula, Muchinga, and Northern provinces of Zambia. The findings of this study may help stakeholders interested in establishing interventions aimed at improving the performance of these chickens, thereby improving the livelihood of rural communities that depend on these chickens for a living.

## 2 Materials and methods

### 2.1 Study location

**Figure 1:**
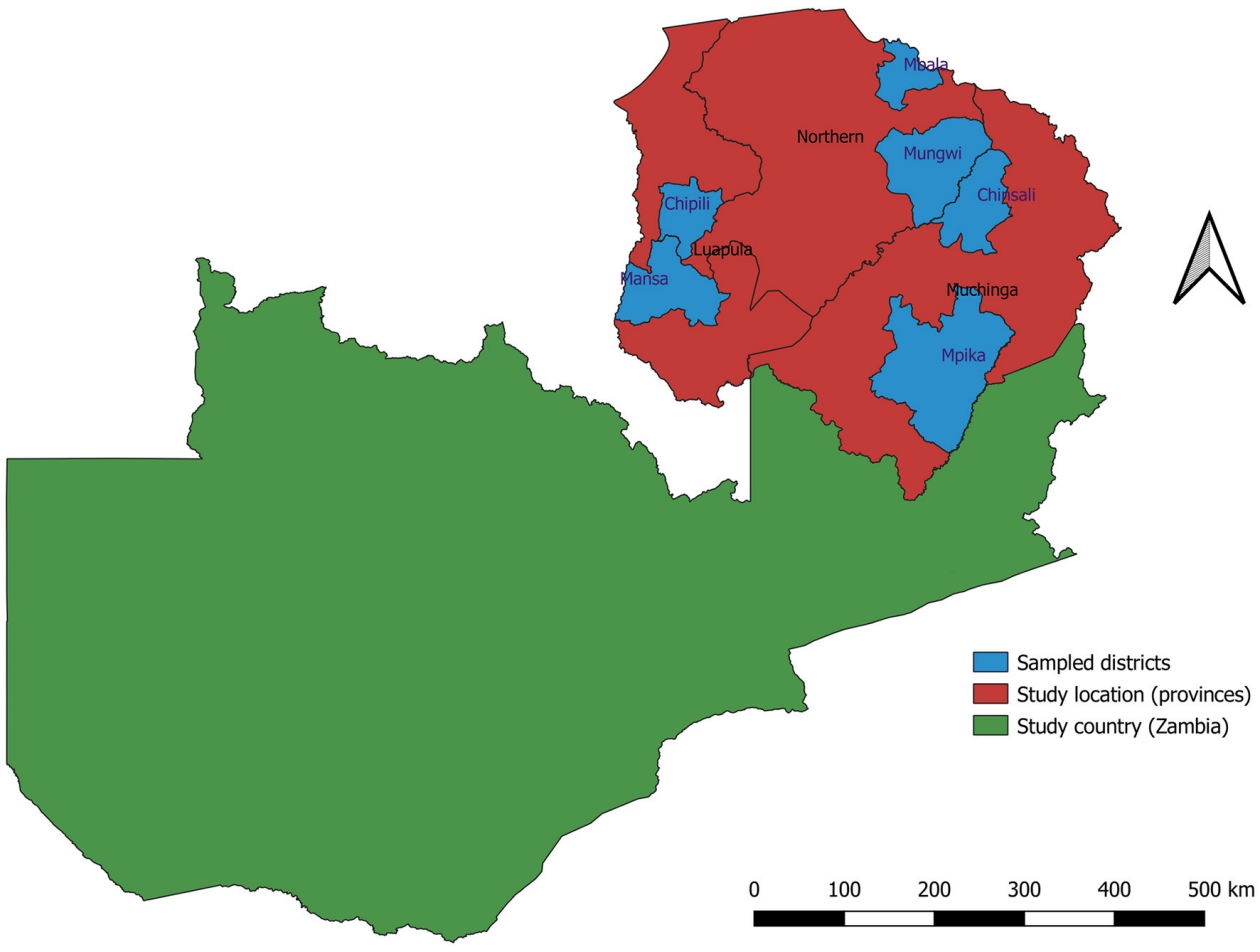
Map of Zambia showing the study area.

Zambia has ten provinces that are divided into three agroecological regions. Distinct climatic characteristics, primarily rainfall, define Zambia's agroecological regions. Agroecological region I, a semi-arid zone in southern, eastern, and western Zambia, receives 600–800 mm of annual rainfall. Agroecological region II, located in central Zambia, benefits from 800 to 1,000 mm of annual rainfall and average temperatures ranging from 23–26°C in October to 16–20°C in June and July. Agroecological region III, encompassing northern Zambia, is a high-rainfall area with over 1,000 mm of precipitation annually. This study covered three (3) provinces: Muchinga, Luapula, and Northern. The three provinces are located in the Northern part of Zambia in agroecological region III. Figure 1 depicts the map of Zambia showing the study area and the sampled districts.

### 2.2 Sampling method and sampling size

A total of 177 indigenous farmers were interviewed. This was the number of farmers with at least five chickens and kept chickens for at least 2 years in the three villages in each agricultural camp sampled per district. For the phenotypic characterizations, 538 chickens were sampled and phenotyped. These comprised 302 hens and 236 cocks.

### 2.3 Data collection procedure

A structured questionnaire was employed to collect information on social demographics, production systems, production performance, breeding management, husbandry practices, marketing, health management practices, and challenges faced by farmers. Data were collected through individual interviews. The production and reproductive performance were based on the farmers' recall and, in a few instances, on farmers' records.

Each chicken was phenotyped for six (6) qualitative traits that included plumage/color, skin color, shank color, comb type, earlobe color, and eye color, which were taken by observing each sampled chicken individually. Nine (9) body measurements were taken using a flexible tailor's measuring tape in centimeters. These were corpus length (CL), chest circumference (CC), thigh length (TL), thigh circumference (TC), shank circumference (SC), Shank Length (SL), Keel length (KL), body length (BL) and the wingspan (WS). The methods used for taking the qualitative and quantitative traits were according to the FAO (16) guide for characterizing animal genetics resources and as applied earlier (4). To reduce the bias effect, all measurements were taken by the same person trained in the same. Body weights were taken using a digital scale with a sensitivity of 10 grams.

### 2.4 Data analysis

All the data were tabulated and edited in the Excel©. MINITAB V 22 software (2020) was used for the statistical analysis. Frequencies were used to present the qualitative results. Analysis of variance (ANOVA) was used for the quantitative traits to compare means between provinces at a 95% confidence interval. Pearson correlation was conducted to assess the association between the linear body measurements and the body weight. This was the basis on which the principal component analysis (PCA) was used to measure the variations in traits and characterizing the indigenous chickens. PCA is a technique used to decrease the dimensionality of a multivariate dataset while retaining the highest possible variance from the initial set of variables, achieved through a minimal number of composite variables. To evaluate the validity of examining factors within the number sets, the researchers performed Bartlett's test of sphericity. Additionally, the suitability of the dataset for analysis was affirmed through the Kaiser-Meyer-Olkin (KMO) measure of sampling adequacy, which assessed whether the correlations among variables were sufficiently low. A KMO value ≥ 0.60 was considered satisfactory.

## 3 Results

### 3.1 Demographics and production systems

#### 3.1.1 Household characteristics

Table 1 displays the socio-demographics of the small-scale farmers of indigenous chickens. This study revealed that most small-scale farmers who keep indigenous chickens are females. The dominance of females in the free-range chicken production was also reflected in Luapula and Muchinga provinces. However, this was different in the Northern Province, where males slightly dominated the production of indigenous chickens.

**Table 1:** Social demographics of small-scale farmers in Luapula, Muchinga, and Northern Province and their motivation for keeping Indigenous chicken.

**Variable**	**Proportion (%)**			
	**Luapula**	**Muchinga**	**Northern**	**Overall**
**Gender**				
Female	67.44	78.64	48.94	65.37
Male	32.56	21.36	51.06	34.63
**Age**				
≤ 30	16.28	8.74	31.91	18.73
31–40	27.91	27.18	14.89	23.32
41–50	17.44	29.13	17.02	21.55
51–60	27.91	27.18	21.28	25.44
>60	10.46	7.76	14.90	10.96
**Education**				
No formal education	11.63	1.94	6.38	6.36
Primary	59.30	63.11	70.21	64.31
Secondary	29.07	33.01	23.40	28.62
Tertiary	0.00	1.94	0.00	0.71
**Source of income**				
Farming	98.84	99.03	95.74	97.88
Informal employment	1.16	0.97	0.00	0.35
Off-farm business	0.00	0.00	4.26	1.77
Household size (Mean ± SE)^a^	6 ± 0.20	7 ± 0.15	6 ± 0.21	6.30 ± 0.11
**Occupation**				
Charcoal burner	0.00	0.00	2.13	0.71
Civil servant	0.00	0.97	0.00	0.35
Farming	100.00	98.06	97.87	98.59
Student	0.00	0.97	0.00	0.35
**Other livestock kept**				
Cattle	0.00	6.80	38.30	15.19
Ducks	2.33	13.59	6.38	7.77
Goat	51.16	56.31	42.55	50.18
Pigs	6.98	13.59	20.21	14.13
Rabbits	6.98	4.85	24.47	12.37
Sheep	3.49	0.00	2.13	1.77
**Reasons for keeping chickens**				
Cash from sale	96.51	96.12	97.87	96.82
Ceremony	10.47	0.97	2.13	4.24
Cultural	11.63	1.94	17.02	9.89
Egg consumption	61.63	83.50	40.43	62.54
For replacement	0.00	0.00	12.77	4.24
Manure	2.33	2.91	25.53	10.25
Meat consumption	89.53	95.15	93.62	92.93

^a^SE means standard error of the mean.

Most people keeping indigenous chickens were aged between 30 and 60 years. A large proportion of them have had primary education. Almost all respondents indicated farming as their main source of income and the most dominant occupation. The household size did not differ significantly (*P* > 0.05) between provinces. The average household size was 6.30 ± 0.11 persons per household (Table 1). Almost all the respondents indicated using traditionally owned land to produce indigenous chickens. Goats were the most commonly kept livestock alongside indigenous chickens in the study area.

The main reasons for keeping chickens were cash from sales, meat, and egg consumption. Other less common reasons included ceremony purposes, cultural reasons, replacement, and manure. This picture was the same across all the provinces (Table 1).

#### 3.1.2 Production system, housing type, and nutrition of indigenous chickens

The free-range system of rearing indigenous chickens was the most popular across the three provinces (Table 2). The analysis from the provinces showed that the most common housing type in Luapula Province was a family house. In the Northern province, the most common housing was designated poultry houses. In Muchinga province, farmers preferred chickens to sleep in trees. Further interrogation showed that trees and family houses were preferred in places with high-security risks from thieves. Hand-woven baskets were another uncommon housing type found in Northern and Muchinga provinces.

**Table 2:** Housing, types of bedding, feeding, and production system of indigenous chickens in Luapula, Muchinga, and Northern province of Zambia.

**Variable**	**Response**	**Luapula (%)**	**Muchinga (%)**	**Northern (%)**	**Overall (%)**
Housing	Chicken house	25.58	33.01	53.19	37.46
	Family house	65.12	28.16	36.17	42.05
	Hand-woven basket	0	4.85	4.26	3.18
	Trees	9.3	33.98	6.38	17.31
Beddings	Chopped grass	1.32	4.04	4.44	3.4
	Crop residues	0	6.06	0	2.26
	None	72.37	70.71	60	67.55
	Sack	25	14.14	31.11	23.02
	Wood shavings/Sawdust	1.32	5.05	4.44	3.77
Feeding	Chickens scavenge for their water and feed	100	100	100	100
	Supplementation	12.79	58.25	61.7	45.58
Feeding procedure	Throw at ground	12.59	54.37	55	42
	Using a container or feeder	0.2	3.88	6.7	3.58
Watering	Provide water and feed	70.93	86.41	78.72	79.15
Production type	Extensive/free range	90.7	80.58	63.83	78.09
	Semi-intensive	9.3	19.42	36.17	21.91

Most respondents did not provide any form of bedding for their chickens (Table 2). However, the most common form of bedding among those that provided bedding was sacks. Other rare bedding forms were chopped grass, crop residues, and wood shavings.

All farmers let their chickens scavenge for their nutrition, with an average of 45.58% supplementing the scavenging with Basic Feeds (Table 2). The supplementation varied from kitchen refuse to broken grains from processing grains for home consumption. The bulk of those who fed their chickens just threw the feed to the ground (Table 2), and only a few used some form of feeders or containers to feed their chickens. The majority of farmers provided water to their chickens.

### 3.2 Breeding management and selection practices

#### 3.2.1 Source of breed stock, mating system, and breeding system

**Figure 2:**
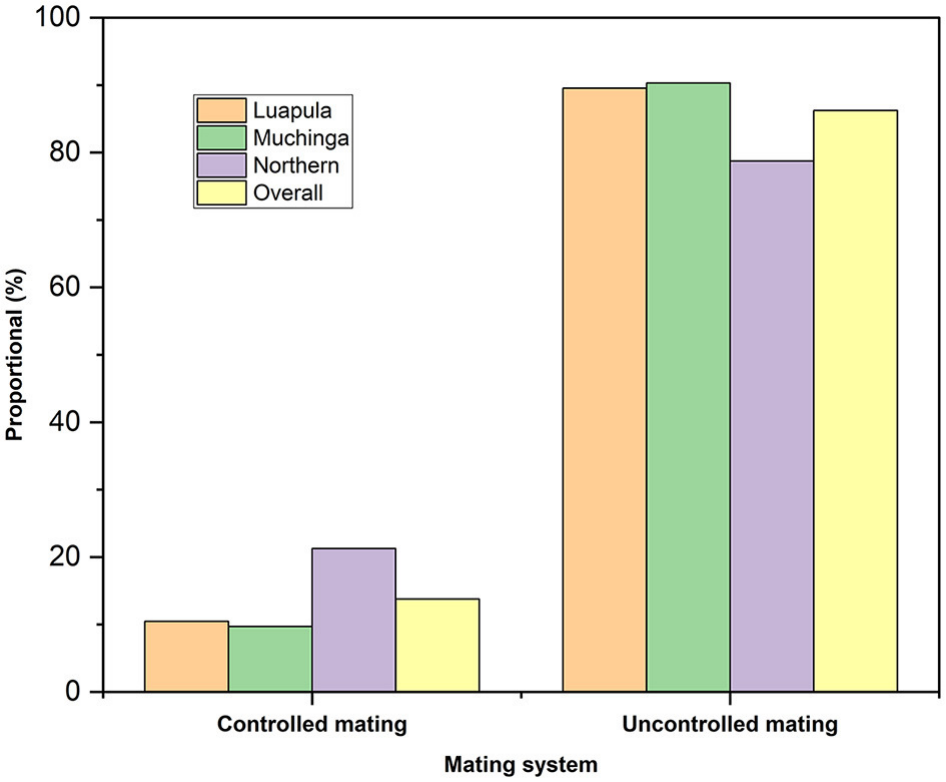
Chicken mating practices among small-scale Indigenous chicken farmers in Muchinga, Luapula, and Northern provinces of Zambia.

**Figure 3:**
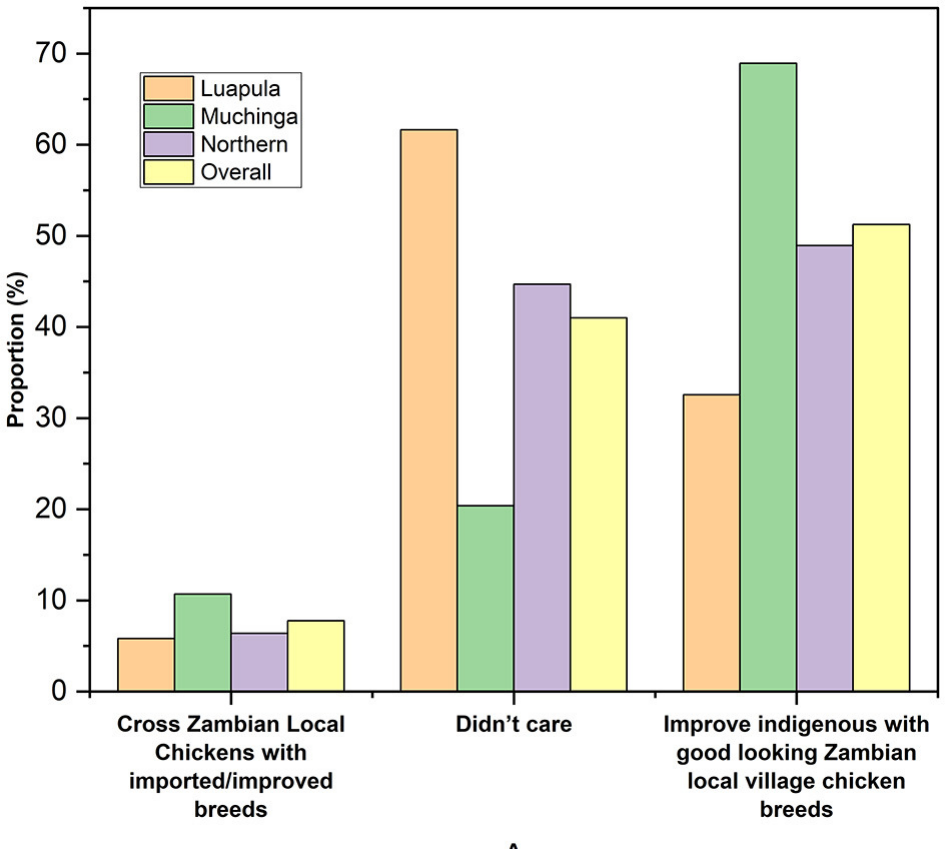
Chicken Breeding practices among small-scale Indigenous chicken farmers in Muchinga, Luapula, and Northern provinces of Zambia.

Table 3 shows the sources of breeding stock by small-scale farmers of indigenous chickens in Luapula, Muchinga, and Northern Provinces. Most households acquired their breeding stocks from within the community. Overall, the second most common source of breeding stock was outside the community and roadside. However, only very few of the respondents bred their breed stock. Some obtained their breed stock by exchanging with neighbors, and few received their breeding stock as gifts. The overall flock size per household was 12.5 ± 0.91 chickens (Table 4). No significant differences (*P* > 0.05) across the three provinces regarding the flock size per household were observed. The present study revealed that most farmers mated indigenous chickens among themselves (Figure 2), and most practiced uncontrolled breeding (Figure 3).

**Table 3:** Sources of breed stock and culling practices among small scale farmers of indigenous chickens in Luapula, Muchinga, and Northern province of Zambia.

**Variable**	**Response**	**Luapula (%)**	**Muchinga (%)**	**Northern (%)**	**Overall (%)**
Source of breed stock	Breed them ourselves	1.16	2.91	2.13	2.12
	Buy from roadside, market etc.	5.81	14.56	8.51	9.89
	Buy from within	87.21	80.58	85.11	84.1
	Exchange with neighbors	0	3.88	2.13	2.12
	Receive as gift	5.81	3.88	4.26	4.59
Culling presence	No	46.51	23.3	0	22.61
	Yes	53.49	76.7	100	77.39
Culling reasons	Bad temperament	0.00	4.85	19.15	8.13
	Illness	47.67	33.01	46.81	42.05
	Low hatchability	44.19	42.72	46.81	44.52
	Low production	88.37	74.76	91.49	84.45
	Old age	59.30	45.63	55.32	53.00
	Poor brooding	0.00	0.97	0.00	0.35
	Unwanted plumage color	6.98	0.00	6.38	4.24
	None of these	0.00	1.94	0.00	0.71
Fate of culls	Home consumption	69.77	78.64	78.72	75.98
	Sale	30.23	20.39	21.28	23.67
	Throw them or burn or bury them	0	0.97	0	0.35

**Table 4:** Reproductive performance of Zambian indigenous chicken in Luapula, Muchinga, and Northern provinces.

	**Mean reproductive performance**				
	**Luapula**	**Muchinga**	**Northern**	***P*-value**	**Overall**
Flock size	12.98	10.30	15.38	0.07	12.50 ± 0.09
Cock age at first mating (months)	6.45^b^	6.55^b^	7.38^a^	0.00	6.80 ± 0.07
Hen age at first mating (months)	6.23^b^	6.16^b^	6.64^a^	0.01	6.34 ± 0.07
Age at first egg (months)	6.66	6.75	6.78	0.54	6.73 ± 0.05
Clutch size (no. of eggs)	13.19	13.41	12.7	0.04	13.11 ± 0.12
Period/clutch (days)	16.08^a^	15.85^a^	14.38^b^	0.00	15.43 ± 0.17
No. of days per egg	1.25^a^	1.19^ab^	1.15^b^	0.00	1.19 ± 001
no. clutches/year	3.25	3.16	3.17	0.48	3.19 ± 0.03
Hatchability (%)	82.46	83.89	83.85	0.56	83.44 ± 0.61
Mortality @ week 8	66.42^b^	72.57^a^	64.26^b^	0.00	67.94 ± 0.90
Market age (months)	7.56	7.11	6.94	0.18	7.23 ± 0.14

Numbers in the same row sharing identical superscripts imply a lack of significant differences (*P* > 0.05), whereas those with distinct superscripts indicate a significant difference (*P* < 0.05).

#### 3.2.2 Culling practices

Table 3 shows the presence of culling practices among indigenous chicken farmers in Luapula, Muchinga, and the Northern provinces of Zambia. Of the households interviewed, the majority of them practiced culling chickens from their flocks.

Low productivity was the most common reason for culling (Table 3). Common reasons for culling included old age, low hatchability, and Illness. This trend was similar in all the provinces. Other uncommon reasons for culling chickens included bad temperament, poor brooding, and unwanted plumage color. As shown in Table 3, home consumption was the most common fate for the culls. Selling of the culls was the second most common fate of the culls. Very few of the households burnt or buried the culls.

### 3.3 Reproductive performance

#### 3.3.1 Age at first mating

Table 4 shows the reproductive performance of Zambian indigenous chickens in Luapula, Muchinga, and Northern provinces. The age of cocks at first mating varied significantly (*P* < 0.05) between provinces and averaged 6.80 ± 0.07 months. In Northern Province, cocks first mated at a significantly (*P* < 0.05) higher age compared to the cocks in Luapula and Muchinga provinces.

Hens first mated at a younger age than the cocks in the study area (Table 4). The age at first mating for hens differed significantly (*P* < 0.05) between the provinces, with Muchinga province having the lowest age at first mating and the Northern province having the highest age at first mating in hens.

#### 3.3.2 Age at first egg, clutch size, and days/egg

The average age of at first egg was taken as the age at sexual maturity of the hens. This study established that despite the age at first mating being 6.34 ± 0.07 months (the age when farmers first observed cocks chasing hens), the age at first egg was 6.73 ± 0.05 months (Table 4). The differences in age at first egg were non-significant (*P* > 0.05) among the provinces.

The Clutch size for the chicken varied significantly (*P* < 0.05) across the three provinces and averaged 13.11 eggs/clutch (Table 4). How long it took to reach the clutch size (clutch length) significantly (*P* < 0.05) varied across the provinces studied and averaged 15.43 days, giving 1.19 days per egg. On average, chickens in the three provinces had just over three clutches per year (Table 4).

#### 3.3.3 Hatchability and mortality

Table 4 shows the hatchability and mortality of chickens in the study area. In the current study, the provinces showed no significant differences in hatchability, with a mean of 83.44%.

Mortality in the first 8 weeks varied significantly (*P* < 0.05) between provinces, averaging 67.94%, giving a survival rate of 32.06%. Muchinga province had the highest, and Northern Province had the lowest mortality rate (Table 4).

### 3.4 Marketing

**Figure 4:**
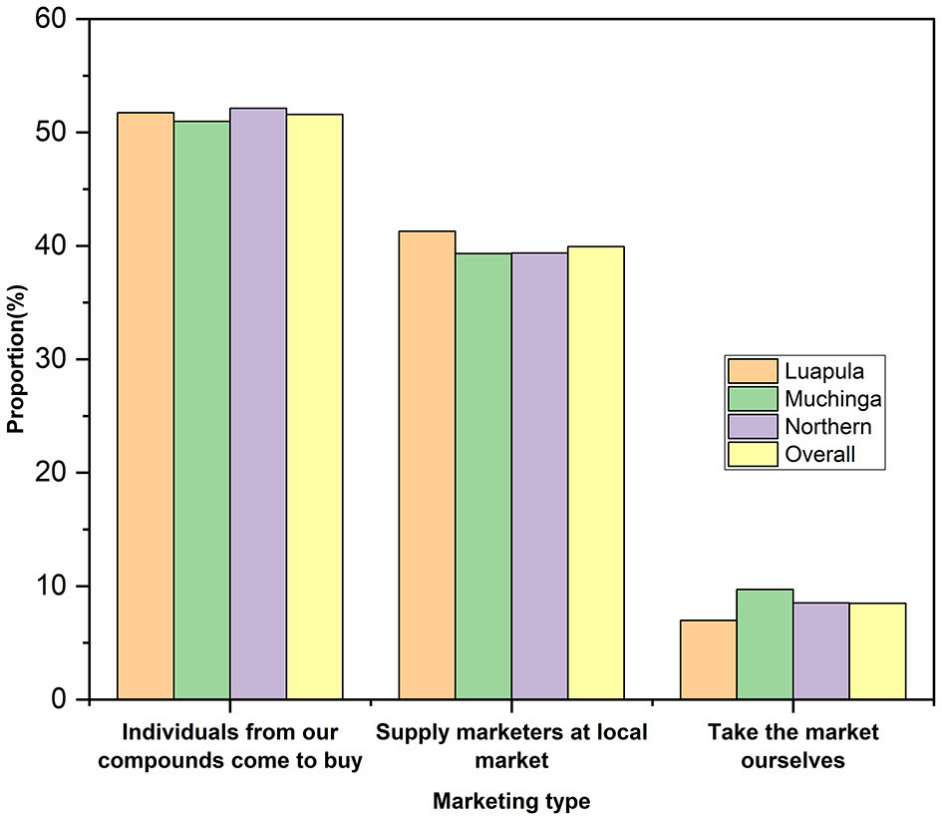
Chicken marketing practices among small-scale Indigenous chicken farmers in Muchinga, Luapula, and Northern provinces of Zambia.

The provinces did not differ significantly (*p* > 0.05) from one another in the marketing age of indigenous chickens in the areas studied, which averaged 7.23 ± 0.14 months (Figure 4)

In all three provinces, most respondents said individuals from within the compounds/villages bought most chickens (Figure 4). The second most common way of marketing was the one where farmers supplied marketers at the local market who later resold the chickens to consumers. All chickens in the three provinces were sold as live birds. No form of value addition was made to the local chickens for marketing.

### 3.5 Challenges of small-scale indigenous chicken farmers

**Figure 5:**
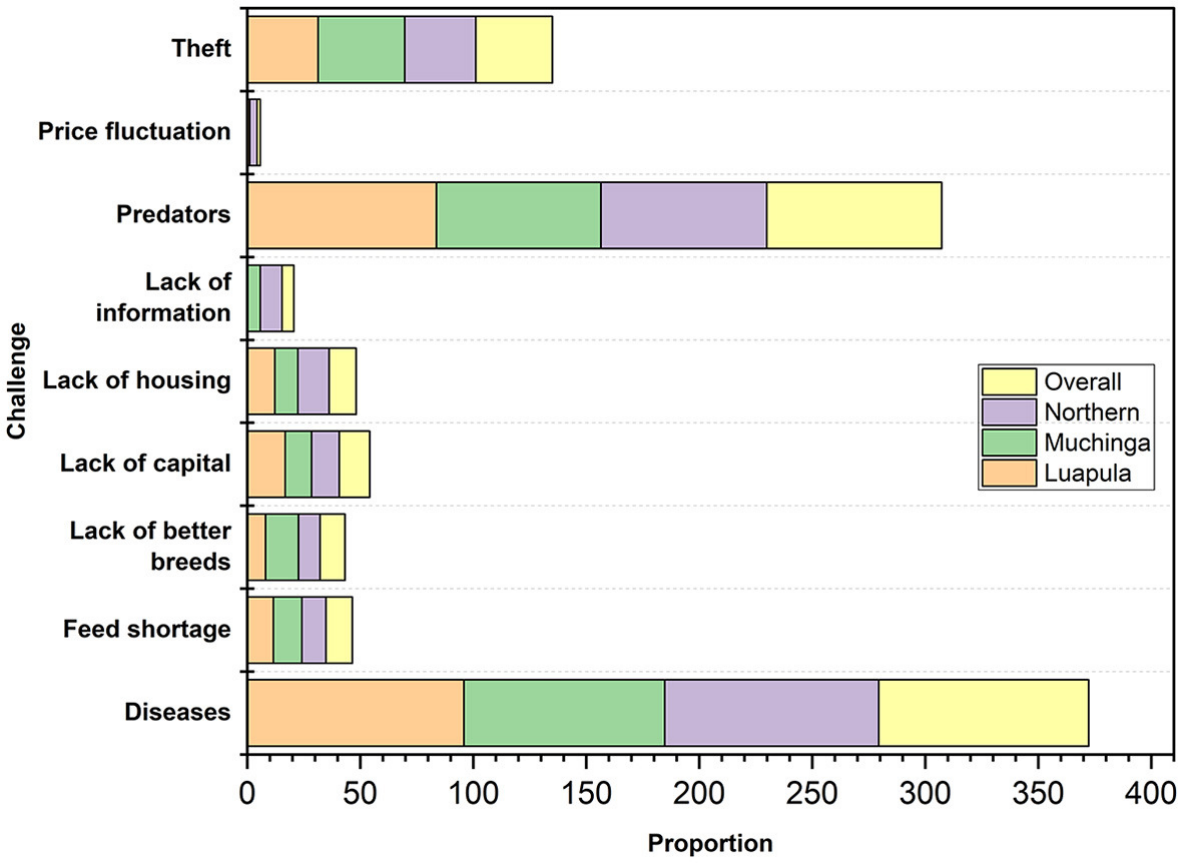
Major challenges faced by small-scale Indigenous chicken farmers in Muchinga, Luapula, and Northern provinces of Zambia.

Figure 5 shows the common challenges faced by small-scale farmers of indigenous chickens in the three provinces. The two most common problems identified were diseases and predators, with theft ranking third. Other challenges included lack of capital, lack of housing, feed shortage, lack of better breeds, lack of information, and price fluctuation.

### 3.6 Diseases and health management practices

Table 5 shows the three provinces' disease and health practices among small-scale free-range chicken producers. The results of this study showed that most farmers in the three provinces never consulted Veterinary or Livestock experts. While most farmers in the three provinces treated their chickens when sick, most neither vaccinated nor dewormed their chickens. However, most of them used traditional remedies to treat their sick chickens.

**Table 5:** Diseases and health management in indigenous chickens in among small scale farmers in Luapula, Muchinga, and Northern Provinces of Zambia.

**Variable**	**Response**	**Luapula**	**Muchinga**	**Northern**	**Overall**
Consults vets	No (%)	69.19	62.23	67.02	65.72
	Yes (%)	30.81	37.77	32.98	34.28
Animal treatments	No (%)	36.05	32.98	33.51	33.57
	Yes (%)	63.95	67.02	66.49	66.43
Any vaccinations	No (%)	54.07	56.38	63.83	57.6
	Yes (%)	45.93	43.62	36.17	42.4
Deworming	No (%)	83.14	79.26	87.77	83.04
	Yes (%)	16.86	20.74	12.23	16.96
Any traditional remedies	No (%)	33.14	19.68	34.57	28.62
	Yes (%)	66.86	80.32	65.43	71.38

### 3.7 Qualitative characterization

Chickens were physically and individually phenotyped. The qualitative traits found in this study are presented in Table 6.

**Table 6:** Qualitative traits of indigenous chickens in Muchinga, Luapula, and Northern Provinces of Zambia.

**Trait**		**Luapula (%)**			**Muchinga (%)**			**Northern (%)**			**Overall (%)**
		**F**	**M**	**ALL**	**F**	**M**	**ALL**	**F**	**M**	**ALL**	
Plumage color	Black	10.14	11.00	10.40	26.92	0.00	19.40	41.10	34.62	38.00	22.68
	Brown	59.42	25.00	44.00	23.08	0.00	16.70	25.00	0.00	13.00	27.88
	Gray	0.00	0.00	0.00	0.00	0.00	0.00	8.93	0.00	4.63	1.86
	Mixed	23.19	43.00	32.00	34.62	19.20	30.60	12.50	26.92	19.40	26.77
	Red	0.00	3.60	1.60	7.69	15.40	11.10	3.57	19.23	11.10	6.69
	White	7.25	18.00	12.00	7.69	65.40	22.20	8.93	19.23	13.90	14.13
Skin color	White	89.86	100.00	94.40	88.46	0	91.70	91.10	84.62	88.00	91.45
	Yellow	10.14	0.00	5.60	11.54	100.00	8.33	8.93	15.38	12.00	8.55
Shank color	Green	15.94	0.00	8.80	15.38	0.00	11.10	30.40	34.62	32.40	18.59
	Gray	1.45	3.60	2.40	38.46	46.20	38.90	32.10	26.92	29.60	18.22
	White	65.22	86.00	74.40	34.62	38.50	36.10	26.80	19.23	23.20	48.70
	Yellow	17.39	11.00	14.40	11.54	15.40	13.90	10.70	19.23	14.80	14.50
Comb type	Cushion	4.35	3.60	4.00	0.00	0.00	0.00	5.36	3.85	4.63	3.72
	Double	0.00	0.00	0.00	0.00	0.00	0.00	1.79	0.00	0.93	0.37
	Rose	4.35	3.60	4.00	0.00	0.00	0.00	7.14	7.69	7.41	4.83
	Single	91.30	93.00	92.00	100.00	100.00	100.00	85.70	88.46	87.00	91.08
Earlobe color	R/W	23.19	7.10	16.00	7.69	38.50	16.70	19.60	19.23	19.40	17.47
	Red	49.28	82.00	64.00	30.77	61.50	38.90	28.60	76.92	51.90	55.76
	White	27.54	11.00	20.00	61.54	0.00	44.40	51.80	3.85	28.70	26.77
Eye color	Brown	4.35	0.00	2.40	26.92	0.00	19.40	19.60	7.69	13.90	9.29
	Orange	68.12	100.00	82.40	73.08	100.00	80.60	64.30	80.77	72.20	78.07
	Pearl	0.00	0.00	0.00	0.00	0.00	0.00	5.36	0.00	2.78	1.12
	Yellow	27.54	0.00	15.20	0.00	0.00	0.00	10.70	11.54	11.10	11.52

#### 3.7.1 Plumage color

A variety of plumage colors of chickens were observed in the three provinces where this study was done (Table 6). Brown chickens were the most common. However, just over a quarter of the chickens had mixed colors. Other plumage colors found were white, red, and gray.

#### 3.7.2 Skin color

The skin colors of the sampled chickens were also examined, and in all three provinces, the most common skin color was white (Table 6). A minority of them had a yellow skin color.

#### 3.7.3 Shank color

White shanks dominated Luapula, while the gray shanks were the most common in Northern and Muchinga provinces. Other shank colors found in the study area are green and yellow shank color.

#### 3.7.4 Comb type

An examination of the comb type revealed that in all the provinces, the single comb type was the most predominant (Table 6). Other comb types found were the cushion, double, and rose type.

#### 3.7.5 Earlobe color

While the red earlobe type dominated in the three provinces (Table 6), sexual dimorphisms were seen in Muchinga and Northern provinces, where white earlobe color dominated in hens, and the red-colored earlobes dominated the cocks, respectively. The red-white combs were also found but in smaller proportions.

#### 3.7.6 Eye color

Most chickens in the provinces studied had orange eyes (Table 6). Other eye colors found included yellow, brown, and pearl.

### 3.8 Quantitative characteristics

#### 3.8.1 Descriptive statistics

Table 7 tabulates the descriptive statistics of the body weight and linear body measurements. A comparison between provinces showed a non-significant difference (*P* > 0.05) in body weight. Numerically, Muchinga province had the heaviest chickens of the three provinces.

**Table 7:** Descriptive statistics (mean values) of the body weight and linear body measurements of indigenous chickens in Muchinga, Luapula, and Northern Provinces of Zambia.

**Trait**	**Luapula**	**Muchinga**	**Northern**	**Mean**	***P*-value**
Weight (kg)	1.64	1.77	1.64	1.66 ± 0.00	0.07
CL (cm)	22.26^a^	22.06^a^	21.10^b^	21.77 ± 0.10	0.00
CC (cm)	28.00^b^	30.20^a^	27.66^b^	28.16 ± 0.15	0.00
TL (cm)	13.28^a^	13.00^ab^	12.87^b^	13.08 ± 0.07	0.02
TC (cm)	8.57^b^	9.44^a^	8.71^b^	8.74 ± 0.08	0.00
SC (cm)	4.53^a^	4.27^b^	4.34^ab^	4.42 ± 0.03	0.00
SL (cm)	9.95	10.02	10.05	10.00 ± 0.07	0.79
KL (cm)	11.72^a^	11.09^b^	11.12^b^	11.40 ± 0.07	0.00
BL (cm)	41.18^b^	43.20^a^	42.44^a^	41.96 ± 0.19	0.00
WS (cm)	42.71^b^	43.26^ab^	44.45^a^	43.48 ± 0.22	0.00

Different superscript means significant differences (*P* < 0.05), same or no superscript means no significant difference (*P* > 0.05).

CL, corpus length; CC, chest circumference; TL, thigh circumference; TC, thigh circumference; SC, shank circumference; SL, shank length; KL, keel Length; BL, body length; WS, wing span.

Linear body measurements differed significantly (*P* < 0.05) among provinces, except for SL (*P* = 0.790). Luapula province recorded the highest CL and TL, while Muchinga province had the lowest CL. CC was notably higher in Muchinga and lowest in the Northern province. TC was highest in Muchinga compared to the other two provinces. Luapula exhibited increased SC compared to Muchinga and the Northern provinces. Muchinga had the lowest KL but the highest BL compared to the other provinces. The Northern province dominated in the size of the WS.

#### 3.8.2 Correlation matrix

The Pearson correlation analysis revealed that all the linear body measurements taken positively and significantly (*P* < 0.05) correlated with the body weight in both males and females (Table 8). All linear body measurements taken in this study positively correlated (*P* < 0.05) with each other, apart from in hens, where a negative relationship existed between wing span and shank circumference was noted.

**Table 8:** Pearson correlation analysis of the body weight and Linear Body measurements Muchinga, Luapula, and Northern Provinces of Zambia (top is female and the bottom is male).

	**Weight**	**CL**	**CC**	**TL**	**TC**	**SC**	**SL**	**KL**	**BL**	**WS**
Weight		0.541^**^	0.492^**^	0.268^**^	0.316^**^	0.248^**^	0.316^**^	0.454^**^	0.432^**^	0.263^**^
CL	0.544^**^		0.224^**^	0.441^**^	0.057	0.497^**^	0.220^**^	0.557^**^	0.326^**^	0.08
CC	0.665^**^	0.445^**^		0.140^*^	0.290^**^	0.01	0.239^**^	0.310^**^	0.313^**^	0.257^**^
TL	0.469^**^	0.351^**^	0.387^**^		0.195^**^	0.282^**^	0.509^**^	0.441^**^	0.316^**^	0.240^**^
TC	0.455^**^	0.117	0.560^**^	0.361^**^		0.054	0.238^**^	0.124^*^	0.301^**^	0.227^**^
SC	0.540^**^	0.562^**^	0.342^**^	0.375^**^	0.01		0.142^*^	0.428^**^	0.143^*^	−0.081
SL	0.518^**^	0.391^**^	0.542^**^	0.498^**^	0.327^**^	0.206^**^		0.357^**^	0.408^**^	0.423^**^
KL	0.380^**^	0.510^**^	0.327^**^	0.339^**^	0.062	0.373^**^	0.539^**^		0.328^**^	0.084
BL	0.668^**^	0.344^**^	0.473^**^	0.471^**^	0.325^**^	0.396^**^	0.463^**^	0.352^**^		0.394^**^
WS	0.507^**^	0.286^**^	0.403^**^	0.385^**^	0.218^**^	0.321^**^	0.477^**^	0.219^**^	0.427^**^	

^*^Correlation is significant at the 0.05 level (2-tailed).

^**^Correlation is significant at the 0.01 level (2-tailed).

CL, corpus length; CC, chest circumference; TL, thigh circumference; TC, thigh circumference; SC, shank circumference; SL, shank length; KL, keel length; BL, body length; WS, wing span.

#### 3.8.3 Principal component analysis

Table 9 shows the principal components, their Eigenvalues, and their commonalities. The KMO measure of sampling appropriateness established in this study on all variables was 0.913 with a highly significant Bartlett's test (*p*-value of 0.000). This warranted a principal component analysis. This study then employed a varimax rotation technique to maximize the variance sum. Only two Principal components with Eigenvalues of more than 1 were extracted, PC1 and PC2, with Eigenvalues of 5.89 and 1.048, respectively. The two PCs cumulatively accounted for the majority of variance, giving a total of 69.38% combined. The communalities established in this study ranged from 0.504 to 0.774, indicating that the model accounted for most variations. PC 1 had lower loading on CC, TC, and WS, while PC 2 had low loadings on CL, TL, SC, and KL. Only body weight, SL, and BL had high loadings on PC1 and PC2. This could mean PC 1 and PC 2 had to do with size.

**Table 9:** Principal Component analysis results.

**Trait**	**Principal component**		**Communality**
	**PC1**	**PC2**	
Weight	0.62	0.624	0.774
CL	0.85	0.248	0.785
CC	0.318	0.739	0.646
TL	0.681	0.447	0.664
TC	0.027	0.847	0.719
SC	0.847	0.118	0.731
SL	0.627	0.561	0.707
KL	0.821	0.27	0.747
BL	0.557	0.592	0.66
WS	0.36	0.612	0.504
Eigenvalue	5.89	1.048	
% variation	58.903	10.477	
Cumulative % variance	58.903	69.38	

CL, corpus length; CC, chest circumference; TL, thigh circumference; TC, thigh circumference; SC, shank circumference; SL, shank length; KL, keel length; BL, body length; WS, wing span; PC, principal component.

## 4 Discussion

Given the subpar performance of the majority of native chicken breeds, it seems advantageous to pursue genetic enhancement by selectively breeding within local chicken populations (17, 18). For breeding programs aimed at improving livestock in a given area to be successful, there is a need for a proper understanding of the production system, health practice, and livestock morphometric characteristics in a targeted area (19–21). In aiming to phenotypically characterize the Zambian indigenous chickens and their production system, this study revealed diversity in farmers' demographics, management, health, and marketing practices. The study also revealed phenotypic diversity in the indigenous chickens in the study area. This diversity forms a good base on which selection and breeding can be based.

Various reasons motivate rural communities' production of indigenous chickens, such as consumption, income generation, cultural and religious reasons, and breeding stock (22). The reasons advanced by farmers in the current study for keeping indigenous chickens are comparable to those reported in Ethiopia (23). This means indigenous chicken rearing is very useful for income and food security at the household level even in Zambia.

Small-scale farmers use various ways to house their indigenous chickens. Some farmers house their chickens in homes, kitchens, perch on trees, and some chicken houses (22). In contrast to Luapula province where the majority housed their chickens within family houses, most farmers in Muchinga and Northern provinces had chicken houses. This is comparable to what was reported in other studies (3).

Indigenous chickens are well-known scavengers and survive on relatively low-quality feeds. The presence of minimal feed supplementation and water provision to indigenous chickens reported in this study is similar to what was reported in other studies (24–26). However, this study revealed that despite most of them giving water and feed, the most common feeding method was throwing to the ground in all the provinces, as reported in Rwanda (22).

The practice of getting rid of unproductive chickens reported in this study is not unique to the Zambian farmers in Muchinga, Luapula, and Northern provinces only, as similar findings have been reported elsewhere (24, 27, 28). Similar to the current study's findings, other researchers reported that the most common reasons for culling chickens in their various studies were low production, old age, low hatchability, and illness (23, 29, 30). This research also found that the fate of most culls was home consumption and selling off, just like it was reported elsewhere (31, 32).

Just like was found in this study, it is common for small-scale farmers elsewhere to obtain their breed stock by buying from within the communities (28, 33). However, comparable to what has been reported before (3), the majority of farmers in the studied provinces of Zambia neither practiced controlled breeding nor controlled mating. Uncontrolled mating is characteristic of small-scale free-range chicken production (27).

The age at first mating for the cocks reported in this study is close to the age at first mating of 6.63 months that was reported in Tanzanian chickens (29). However, this was slightly lower than that reported in South Africa (34) but higher than the 5.76 months reported in the Amhara region of Ethiopia (35). These differences may be attributed to differences in the genetics of the birds as well as management and environment.

Age at first egg is an important trait that can be used as selection criteria because it positively influences growth and egg production (36). The average age at first egg reported in this study is lower than in Tanzania (29, 34). Nonetheless, the age at first egg reported in the present study was higher than that reported in Ethiopian chickens (37, 38). Mwalusanya et al. (39) reported that under free-range farmer management of indigenous chickens, the age at first egg ranges between 6 and 8 months. The differences in the age at first egg reported in this study and that reported by other researchers could be attributed to genetics and non-genetic factors such as nutrition.

The clutch size of 13.11 eggs/clutch reported in this study agrees with the findings in the literature (34). However, the finding of this study is lower than the one reported by other researchers (29, 40) but higher than what Ekeocha et al. (41) and Marwa et al. (42) reported. While this study established that it takes 1.19 days/egg, other studies reported higher values (34, 43). The just over three clutches per year reported in this study agree with the findings of other researchers as reviewed by Macharia et al. (44).

The results of this study on hatchability are akin to what is reported in the literature by Ngogo et al. (29). However, these results conflict with the 79.9% reported by Ayalew et al. (35) and the 55% reported in the FUNAAB alpha chicken by Bamidele et al. (45). Differences in management, genetics, and nutrition may be implicated in the causes of these contradictions in the results on hatchability.

The survivability of 32.44% reported here is lower than what Mengesha et al. (46) reported. Biswas et al. (47) attributed the low survivability of local chickens to predation and disease prevalence, which can be devastating among small-scale farmers, accounting for up to 100% of losses (48).

The age at marketing reported in this study is lower than the market age of 9.16 months reported by Ayalew et al. (35). However, the age at marketing recorded in this study was slightly lower than the 8–12 months range reported by Matawork (6). Nonetheless, age at marketing is subject to genetics and environmental factors.

The challenges faced by small-scale farmers of indigenous chickens in the studied areas of Zambia are not unique but are common elsewhere. Diseases have been reported to be one of the biggest problems by Ngongolo and Chota (48), accounting for up to 100% of losses. Predators have also been reported to be a big challenge among indigenous chicken farmers elsewhere (29, 31, 34). Thieves, lack of capital, lack of housing, predators, feed shortage, lack of information, and market price fluctuation are also not unique to Zambia as they have been reported extensively in literature as being challenges elsewhere (3, 49–53).

Like in Zambia, using traditional remedies in indigenous chickens is prevalent among small-scale farmers in indigenous chickens elsewhere. Ayalew et al. (35) reported using *Feto and Damakessie* as ethno-veterinary medicines to treat diseases in indigenous chickens. Niger small-scale farmers of indigenous chickens have been reported to use traditional remedies in treating diseases (54).

Live body weight in chickens is an economically important trait (55) that is affected by several factors among them heat stress (56). The average body weight of the chickens in the studied area was lower than other Zambian indigenous chickens studied in Kalomo, South of Zambia, which weighed an average of 1.97 kg (4, 57). While low body weight is characteristic of indigenous chickens, higher weights of up to 3 kgs have been reported in indigenous chickens (58). The Lower weight recorded in the study may suggest that these chickens have not undergone genetic mixing (4, 59) and, therefore, have the potential for improvement.

As reported in this study, other studies have also reported a positive correlation between linear body measurements and body weight in indigenous chickens (4, 27, 55, 57, 58). The positive relation indicates that selection for any of these traits would result in a corresponding increase in body weight.

Bartlett's tests on the data used in this study were highly significant (*P*-value = 0.000), making this data suitable for PCA. The study had a KMO value of 0.913, way above the 0.60 above which the sample is considered adequate (60, 61).

The varimax rotation employed in this study extracted two principal components, PC1 and PC2, with a combined variance of 69.38% of the total variation. The remainder of the variations can be attributed to the separation of random alleles at contributing genetic locations, inaccuracies in measurement, and environmental factors (62). Other studies have also reported two PCs in chickens (63, 64). The variables that had a heavy loading of the first component (Weight, CL, TL, SC, SL, KL, and BL) tend to describe PC1 as Size and shape, just as reported by Selvan et al. (65).

The shared variance between variables is represented by the commonalities obtained after extracting principal components (66). In this study, the body weight and all linear body measurements had high communalities, indicating that the majority of the variations were accounted for in the study.

## 5 Conclusion

Overall, this research underscores the importance of indigenous chickens in Zambia's rural communities and highlights the need for targeted interventions to improve their management practices, genetics, and productivity. By addressing the challenges faced by small-scale farmers and implementing sustainable breeding programs, these indigenous chickens can play an even more significant role in alleviating poverty, enhancing food security, and contributing to rural development in Zambia. The diversity in phenotypes and the results of the multivariate analysis reveal the potential these chickens have to respond to selective breeding.

## Data availability statement

The raw data supporting the conclusions of this article will be made available by the authors, without undue reservation.

## Ethics statement

The studies involving humans were approved by Mulungushi University School of Medicine and Health Sciences Ethics Review Committee. The studies were conducted in accordance with the local legislation and institutional requirements. Written informed consent for participation was not required from the participants or the participants' legal guardians/next of kin because most respondents had low literacy levels, as such verbal consents were more appropriate as guided by local authorities in the study areas who were witnesses to the study.

## Author contributions

SL: Formal analysis, Investigation, Methodology, Writing – original draft, Writing – review & editing. KM: Conceptualization, Supervision, Writing – review & editing, Project administration. TT: Methodology, Visualization, Writing – review & editing, Formal analysis. KO: Methodology, Validation, Visualization, Writing – review & editing. MM: Investigation, Software, Visualization, Writing – review & editing. XS: Data curation, Software, Visualization, Writing – review & editing. RX: Conceptualization, Funding acquisition, Project administration, Resources, Supervision, Writing – review & editing, Methodology. NQ: Data curation, Funding acquisition, Methodology, Resources, Validation, Writing – review & editing, Investigation.
